# Addition of a Novel, Protective Family History Category Allows Better Profiling of Cardiovascular Risk and Atherosclerotic Burden in the General Population. The Asklepios Study

**DOI:** 10.1371/journal.pone.0063185

**Published:** 2013-05-02

**Authors:** Caroline M. Van daele, Tim De Meyer, Marc L. De Buyzere, Thierry C. Gillebert, Simon L. I. J. Denil, Sofie Bekaert, Julio A. Chirinos, Patrick Segers, Guy G. De Backer, Dirk De Bacquer, Ernst R. Rietzschel, on behalf of the Asklepios Investigators

**Affiliations:** 1 Department of Cardiovascular Diseases, Ghent University Hospital, Ghent, Belgium; 2 Department of Mathematical Modelling, Statistics and Bioinformatics, Faculty of Bioscience Engineering, Ghent University, Ghent, Belgium; 3 Bimetra, Clinical Research Center Ghent, Ghent University Hospital, Ghent, Belgium; 4 Philadelphia VA Medical Center and University of Pennsylvania, Philadelphia, Pennsylvania, United States of America; 5 Institute of Biomedical Technology (IbiTech), Ghent University, Ghent, Belgium; 6 Department of Public Health, Faculty of Medicine and Health Sciences, Ghent University, Ghent, Belgium; Innsbruck Medical University, Austria

## Abstract

**Objectives:**

Whereas the importance of family history (FH) is widely recognized in cardiovascular risk assessment, its full potential could be underutilized, when applied with its current simple guidelines-based definition (cFH): presence of premature cardiovascular disease (CVD) in a first-degree relative. We tested the added value of a new, extended family history definition (eFH), also taking into account later onset of disease, second-degree relatives and number of affected relatives, on profiling cardiovascular risk and atherosclerotic burden in the general population.

**Design:**

longitudinal population study.

**Setting:**

random, representative population sample from Erpe-Mere and Nieuwerkerken (Belgium, primary care).

**Subjects:**

2524 male/female volunteers, aged 35–55 years, free from overt CVD.

**Main outcome measures:**

Subjects were extensively phenotyped including presence of atherosclerosis (ultrasound) and a newly developed FH questionnaire (4 generations).

**Results:**

Compared to cFH, eFH was superior in predicting an adverse risk profile (glycemic state, elevated blood pressure, lipid abnormalities, presence of metabolic syndrome components) and presence of atherosclerosis (all age & sex-adjusted p<0.05). Unlike cFH, eFH remained a significant predictor of subclinical atherosclerosis after adjusting for confounders. Most relations with eFH were not graded but showed clear informational breakpoints, with absence of CVD (including late onset) in any first-degree relative being a negative predictor of atherosclerosis, and a particularly interesting phenotype for further study.

**Conclusions:**

A novel, extended FH definition is superior to the conventional definition in profiling cardiovascular risk and atherosclerotic burden in the general population. There remain clear opportunities to refine and increase the performance and informational content of this simple, readily-available inexpensive tool.

## Introduction

Cardiovascular disease (CVD) aggregates in families [1], [2], [3], [4]. Family history (FH) represents the integration of risk within a family from shared genetic susceptibilities and familial clustering of environmental exposures, lifestyles and behaviours [5]. Accurately defining FH of CVD will have increasing importance in the prevention and treatment of CVD in the post-genome era [6], [7], [8]. Although the term FH is frequently used, there is no common definition [8]. Nearly all definitions are assessments of either “any FH of CVD” or “CVD history in a first-degree relative” and are usually treated as a simple binary variable according to the occurrence or non-occurrence of disease [9], [10]. The current most common definition used in guidelines (cFH) is occurrence of premature CVD (<55 years for men and <65 years for women) in a first-degree relative [11]. Taking into account additional elements could extend the information content of “family history”. Multiple approaches attempting to define which are the key elements of FH have been studied, including: age at onset (premature, late occurrence of disease), degree of relationship (first, second-degree), type of relative (sibling, parent), number of affected relatives and lineage (maternal, paternal) [10], [12], [13], [14].

In first-degree relatives coronary heart disease (CHD) risk is greater given younger ages of onset, but -to a lesser extent- also late-onset CHD is associated with early-onset CHD in the proband [10], [12], [13]. Furthermore, sibling history of CHD might be a stronger risk factor than parental history [10], [15], [16]. CHD in second-degree relatives is associated with early-onset CHD in the proband, especially with more than one affected relative or with early-onset disease [12]. Increased CHD risk is associated with increasing numbers of first- and second-degree relatives with CHD [10], [12]. With regard to lineage the evidence for differential transmission of CHD is far from uniform [10], [12], [13], [17].

Taking into account these key additional elements, we propose a novel, extended FH definition (Asklepios eFH) and define its additional value in describing the risk factor profile and presence of subclinical cardiovascular damage in a large representative population sample.

## Methods

### Ethics Statement

The study complies with the declaration of Helsinki, the protocol was approved by the ethical committee of the Ghent University Hospital and all subjects gave written informed consent.

### Study Population

Subjects were derived from the Asklepios Study, an extensively phenotyped population-representative random sample of 2524 male/female volunteers aged 35–55 years, from the Belgian communities of Erpe-Mere and Nieuwerkerken, free from clinically overt CVD at baseline. An in-depth description of the ASKLEPIOS study protocol has been published [18].

Exclusion criteria were: 1. clinical presence of atherosclerosis/atherothrombosis; 2. major concomitant illness; 3. Diabetes mellitus (DM) type 1, and type 2 if proven macro-vasculopathy or significant renal impairment; 4. conditions precluding accurate haemodynamic assessment (atrial fibrillation, pregnancy); 5. inability to provide informed consent [18].

### Participant Examination: Overview

After obtaining written informed consent, review of questionnaire data and rest, measurements included: basic clinical data, blood sampling and cardiac and vascular echography. All measurements were single observer. Blood pressure (BP) was recorded using cuff-patient matched bilateral triplicate measurements on a sitting subject using a validated oscillometric device (Omron HEM-907). Body mass index (BMI) was calculated as weight (kg)/height (m)^2^. Metabolic syndrome (MS) was defined according to the revised ATP-III criteria [19].

### Biochemical Analyses

All subjects were fasting, had refrained from smoking for at least 6 hours and were screened for intercurrent infection/inflammation before blood sampling (in which case blood sampling was postponed). Serum parameters were measured on a Modular P automated system (Roche Diagnostics, Mannheim, Germany), in an ISO 9002 certified reference laboratory. Impaired fasting glycemia (IFG) denotes a fasting glucose level ≥100 mg/dl and <126 mg/dl (diabetes). High-sensitive C-reactive protein (hs-CRP) concentrations were measured by a high-sensitive, particle-enhanced immunoturbidimetric method (Roche Diagnostics, Mannheim, Germany) [20]. Coefficient of variation (CV) of all tests described above was <3.0%. Serum oxidized low-density lipoprotein concentration was measured by a sandwich enzyme-linked immunosorbent assay (Mercodia, Uppsala, Sweden) [21], [22]. Total CV was <7.4%.

### Subclinical Cardiovascular Damage

Carotid and femoral arteries were carefully scanned bilaterally for the presence of plaque (focal protrusion >50% compared to adjacent sites, absolute thickness >1.5 mm). Intima-media thickness (IMT) was defined as the distance from the leading edge of the lumen-intima interface to the leading edge of the media-adventitia interface, measured in end-diastole, at the far wall, 1–2 cm before the bifurcations [23]. Intra-observer coefficient of variation was 5.2% [24]. Atherosclerosis was defined as a carotid or femoral IMT ≥0.9 mm and/or presence of carotid or femoral plaque.

### Family History

The Asklepios FH Questionnaire (see Questionnaire S1), created for this study, provided data on the occurrence of CVD in 4 generations of the respondent’s genetic family (parents, grandparents, siblings and offspring). As the participants had several days to complete the questionnaire, they could obtain additional information from family members. The study nurse together with the subject reviewed the questionnaire during the visit. For all family members, respondents provided the year of birth and the year and cause of death. The questionnaire further queries for the occurrence of fatal and nonfatal CVD events: myocardial infarction, coronary revascularisation, peripheral vascular intervention of inguinal or lower limb arteries, stroke, carotid revascularisation or sudden cardiac death.

**Figure 1:**
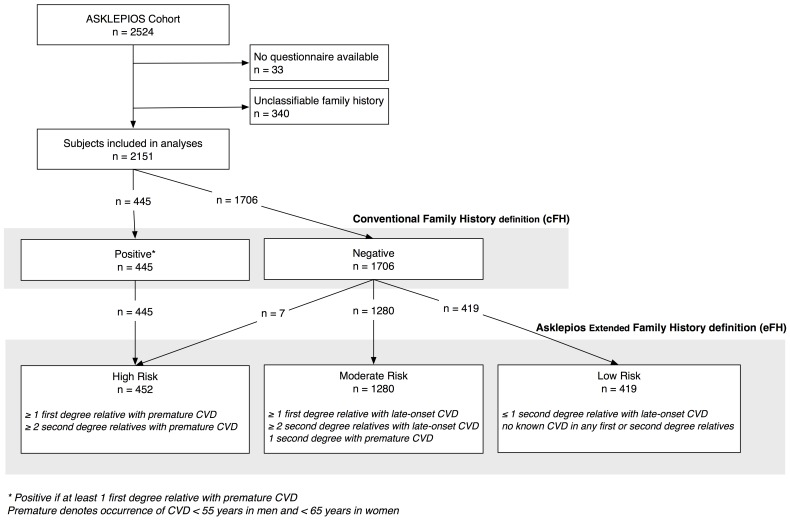
Overview of the distribution of subjects according to the conventional and to the proposed new extended family history definitions. The graph shows the distribution of participants according to the conventional, guidelines-based definition (cFH) and the proposed new extended Asklepios family history definition (eFH). The new eFH high-risk group is almost identical to the guidelines-defined cFH positives. Seven subjects, categorized as negative in the cFH were categorized as high risk in the eFH (those having at least two second-degree relatives (grandparents) with premature CVD). The new eFH definition mainly differs from the conventional definition (cFH) by sub-stratifying the cFH negative group into two categories in the eFH: a large moderate-risk subgroup and a smaller low-risk subgroup.

We propose a more comprehensive extended FH construct and divided participants into 3 categories according to their FH: high, moderate and low risk (Fig. 1) [25]. The eFH definition takes into account additional elements such as age at onset of disease (premature, late occurrence), degree of relationship (first, second-degree (grandparents)) and number of affected relatives. The high-risk category was based on literature review and practice guidelines [11], [12]. The low-risk category was adapted from literature and a stratification model from Scheuner et al. used to address common chronic diseases in a prenatal setting and subsequently shown to be useful in assessing FH in internal medicine [6], [10], [12], [13], [26]. All other subjects where categorized as moderate risk.

### Statistical Analysis

Statistical analysis was performed using SPSS Statistics 20.0. (SPSS Inc., Ill. USA) and R (R-2.15.3, www.r-project.org). In Table 1, we compared the anthropometric, biochemical, metabolic, lifestyle, and other classic cardiovascular risk factors in the different FH categories. As most of these variables were continuous variables, we used age- and sex-adjusted general linear models (GLM) and data are reported as estimated marginal means (95% confidence interval). For categorical variables, the differences between the FH risk categories were calculated by using chi-square tests.

**Table 1:** Age- and sex-adjusted analyses on the new Asklepios extended family history definition (eFH) and the conventional family history definition (cFH).

	New extended family history definition (Asklepios eFH)			Test statistic *p*				Conventional family history definition (cFH)		
*Variable*	Low risk	Moderate risk	High risk	Overall	Low vs Moderate risk	Low vs High risk	Moderate vs high risk	Negative FH	Positive FH	Test statistic *p*
	n = 419	n = 1280	n = 452					n = 1706	n = 445	
Age (years)*	44.2 (43.6-44.8)	46.4 (46.0-46.7)	46.2 (45.7-46.8)	<0.001				45.8 (45.5-46.1)	46.2 (45.7-46.8)	0.22
Sex (% male) †	47.8 (42.9-52.6)	48.1 (45.3-50.8)	46.6(42.0-51.2)	0.86				48.0 (45.6-50.3)	46.6 (42.0-51.3)	0.62
Height (cm)	169.3 (168.7-169.9)	169.1 (168.8-169.4)	169.3 (168.8-169.9)	0.75	0.58	0.94	0.51	169.1 (168.8-169.4)	169.3 (168.7-169.9)	0.66
Weight (kg)	73.1 (71.9-74.3)	73.7 (73.0-74.4)	74.9 (73.8-76.0)	0.072	0.34	0.038	0.071	73.6 (73.0-74.2)	74.9 (73.7-76.0)	0.048
Body Mass Index (kg/m^2^)	25.3 (25.0-25.7)	25.6 (25.4-25.9)	26.0 (25.6-26.4)	0.048	0.164	0.018	0.095	25.6 (25.4-25.8)	26.0 (25.6-26.4)	0.045
Waist circumference (cm)	85.7 (84.7-86.7)	86.4 (85.8-87.0)	87.9 (87.0-88.8)	0.004	0.178	0.002	0.010	86.2 (85.7-86.7)	87.8 (86.9-88.8)	0.004
**Metabolic, biochemical, inflammatory parameters**										
Total cholesterol (mmol/l)	5.5 (5.4-5.6)	5.6 (5.6-5.7)	5.6 (5.6-5.7)	0.062	0.018	0.128	0.96	5.60 (5.56-5.65)	5.64 (5.55-5.72)	0.52
(mg/dl)	213.0 (209.5-216.4)	217.6 (215.6-219.5)	217.7 (214.4-221.0)					216.4 (214.7-218.1)	217.6 (214.3-221.0)	
LDL-cholesterol (mmol/l)	3.30 (3.21-3.38)	3.40 (3.35-3.46)	3.44 (3.36-3.52)	0.042	0.024	0.052	0.46	3.37 (3.33-3.42)	3.44 (3.36-3.52)	0.166
(mg/dl)	127.3 (124.1-130.5)	131.3 (129.5-133.5)	132.7 (129.6-135.8)					130.3 (128.7-131.9)	132.8 (129.7-135.9)	
Non-HDL-Cholesterol (mmol/l)	3.84 (3.75-3.93)	3.98 (3.93-4.03)	4.04 (3.95-4.13)	0.005	0.005	0.008	0.26	3.94 (3.90-3.99)	4.04 (3.95-4.13)	0.054
(mg/dl)	148.1 (144.6-151.7)	153.6 (151.6-155.7)	155.9 (152.6-159.3)					152.3 (150.5-154.0)	156.0 (152.6-159.4)	
HDL-cholesterol (mmol/l)	1.68 (1.64-1.72)	1.66 (1.63-1.68)	1.60 (1.56-1.63)	0.008	0.28	0.006	0.011	1.66 (1.64-1.68)	1.60 (1.56-1.63)	0.002
(mg/dl)	64.9 (63.3-66.3)	63.9 (63.1-64.8)	61.7 (60.3-63.1)					64.2 (63.4-64.9)	61.6 (60.1-63.1)	
Ln Triglycerides (mmol/l)	0.002 (-0.046-0.051)	0.079 (0.052-0.107)	0.121 (0.075-0.168)	0.002	0.007	0.001	0.130	0.061 (0.037-0.085)	0.121 (0.074-0.167)	0.026
(mg/dl)	4.49 (4.44-4.53)	4.56 (4.53-4.59)	4.60 (4.56-4.65)					4.54 (4.52-4.57)	4.60 (4.56-4.65)	
Lipid-lowering therapy (%)	5.6 (3.2-8.0)	6.9 (5.6-8.3)	6.9 (4.6-9.1)	0.62	0.36	0.38	0.98	6.6 (5.4-7.7)	7.0 (4.7-9.3)	0.75
Glycemic state (%)										
Euglycemic	89.7	84.4	84.1					85.8	83.8	
IFG ≥ 100 mg/dl	9.5	14.3	13.5	0.009	0.024	0.048	0.198	13.1	13.7	0.071
Type 2 diabetes	0.7	1.3	2.2					1.2	2.2	
Number of MS components (%)										
0	47.8	35.6	32.6					38.4	33.1	
1	31.1	32.0	33.3	<0.001	<0.001	<0.001	0.71	32.0	32.4	0.24
2	11.7	19.9	19.7					17.9	19.8	
≥3	9.4	12.5	14.4					11.7	14.7	
Oxidized LDL-cholesterol (U/l)	91.6 (88.1-95.4)	96.4 (94.3-98.5)	99.8 (96.3-103.3)	0.008	0.021	0.003	0.111	95.3 (93.5-97.1)	99.8 (96.2-103.3)	0.026
Fibrinogen (mg/dl)	318 (312-324)	326 (322-329)	328 (322-333)	0.055	0.026	0.027	0.55	324 (321-327)	327 (322-333)	0.28
Ln hs-CRP (mg/dl)	0.23 (0.13-0.34)	0.24 (0.18-0.30)	0.25 (0.15-0.36)	0.96	0.90	0.88	0.78	0.24 (0.18-0.29)	0.25 (0.15-0.36)	0.80
**Blood pressure**										
Systolic BP (mmHg)	124.8 (123.5-126.0)	127.1 (126.3-127.8)	128.5 (127.2-129.7)	<0.001	0.002	<0.001	0.054	126.5 (125.9-127.1)	128.4 (127.2-129.6)	0.007
Diastolic BP (mmHg)	78.6 (77.6-79.5)	80.0 (79.5-80.6)	81.4 (80.5-82.3)	0.001	0.005	<0.001	0.008	79.7 (79.2-80.2)	81.3 (80.5-82.2)	0.001
Drug-treated hypertension (%)	7.5 (4.6-10.4)	11.1 (9.4-12.7)	12.0 (9.1-14.7)	0.067	0.035	0.019	0.64	10.2 (8.7-11.6)	12.1 (9.2-14.9 )	0.24
**Lifestyle**										
Daily fruit and vegetable intake (g)	349 (334-364)	338 (330-347)	342 (327-356)	0.49	0.25	0.53	0.68	341 (333-348)	342 (327-356)	0.88
Physical activity (>3.5MET; 0,5x/week, %)	37 (33-42)	36 (34-39)	34 (29-38)	0.51	0.75	0.31	0.30	37 (34-39)	34 (29-38)	0.24
Ever smoker (%)	44 (39-49)	49 (46-52)	50 (45-54)	0.173	0.079	0.112	0.75	48 (45-50)	50 (46-55)	0.36
**Global risk calculation**										
Ln 10-year SCORE risk (Belgium; current age; %)	-0.69 (-0.73- -0.65)	-0.59 (-0.62- -0.57)	-0.56 (-0.59- -0.52)	<0.001	<0.001	<0.001	0.093	-0.62 (-0.64- -0.60)	-0.55 (-0.59- -0.52)	0.004

LDL = low-density lipoprotein; HDL = high-density lipoprotein; MS = metabolic syndrome; hs-CRP = high sensitive C-reactive protein; BP = blood pressure; MET = metabolic equivalent of task; IFG ≥100 mg/dl = impaired fasting glycemia (fasting glucose level ≥100 mg/dl and <126 mg/dl). Data are age- and sex-adjusted estimated marginal means (95%-confidence intervals). Continuous variables were tested using general linear modelling; categorical variables were tested using chi-square test.

* Sex-adjusted.

† Age-adjusted.

We used logistic regression to calculate the adjusted odds ratios of having atherosclerosis, according cFH and eFH classes (first age- and sex adjusted and subsequently multivariate adjusted using confounders age, sex, total cholesterol, HDL-cholesterol, systolic BP, smoking, DM and BMI). The level of significance was set at p<0.05; we used a p<0.025 when comparing the eFH classes to account for multiple testing.

Risk models for presence of atherosclerosis were compared using the PredictABEL package within R (version 1.2–1, July 2012) [27], [28], [29]. As the high-risk categories are near identical in the Asklepios eFH and cFH definitions, the analysis was only meaningful in the cFH negative group in which we compared a baseline model including classic cardiovascular risk factors to a model to which the novel Asklepios eFH definition was added. The baseline risk model included age, sex, total cholesterol, HDL-cholesterol, smoking, BMI and diabetes mellitus. We assessed continuous net reclassification improvement (NRI) and integrated discrimination improvement (IDI). Since there are no meaningful risk categories for the presence of atherosclerosis, we calculated the continuous NRI, which is the most objective measure of improvement in risk prediction and can be used universally.

## Results

The FH questionnaire was completed by 2491 out of 2524 Asklepios subjects (Fig. 1). For 2151 out of 2491 subjects (86.4%) all necessary information was available to evaluate FH. We excluded 340 subjects (13.6%) who could not be correctly classified because of insufficiently accurate knowledge of their FH. Basic characteristics and risk factor profile of this unclassifiable group were similar to the overall population, except for a higher BMI (26.5 kg/m^2^ versus 25.8 kg/m^2^).

According to the cFH definition, 1706 subjects (79.3%) had a negative FH and 445 (20.7%) a positive FH. The Asklepios eFH classification categorized 419 subjects (19.5%) as low, 1280 (59.5%) as moderate and 452 (21.0%) as high risk.

The new eFH high-risk group is almost identical to the guidelines-defined cFH positives (intraclass correlation coefficient 0.995). Seven subjects, categorized as negative in the cFH were categorized as high risk in the Asklepios eFH (those having ≥2 second-degree relatives (grandparents) with premature CVD). The new eFH definition essentially differs from the cFH definition by sub-stratifying the cFH negative group into two categories in the eFH: a large moderate risk subgroup and a smaller low risk subgroup (Fig. 1).

### Novel Asklepios eFH Versus Guidelines-defined cFH

Age and sex-adjusted analyses according to eFH and cFH on anthropometric, biochemical, metabolic, lifestyle, and other classic cardiovascular risk factors are shown in Table 1.

In age and sex-adjusted GLM analyses, a FH of CVD (using either definition) was associated with significantly higher BMI, systolic and diastolic BP, triglycerides, oxidized LDL-cholesterol, 10-year CVD risk (SCORE) and a significantly lower HDL-cholesterol.

Furthermore the novel eFH definition (but not cFH) showed significant associations with glycemic state, non-HDL-cholesterol, LDL-cholesterol, the number of MS components, and it showed borderline significant associations with total cholesterol and fibrinogen.

We subsequently assessed 2×2 comparisons (using age and sex-adjusted GLM) of the three categories of the novel eFH (low, moderate and high risk; Table 1). Most of the additional information when using the novel eFH definition can be explained by the newly defined low risk category (Table 1). The risk profile of the eFH moderate-risk group is not that different from the eFH high-risk group and the near-identical cFH positive group. Therefore, the lower risk profile in the cFH negative group (consisting of eFH low+moderate-risk groups), seems to be mainly driven by the admixture of the eFH low-risk component *(* Table 1; total, LDL- and non-HDL-cholesterol, triglycerides, glycemic state, number of MS components, oxidized LDL-cholesterol, systolic BP and 10-year CVD risk).

### Subclinical Vascular Damage

**Figure 2:**
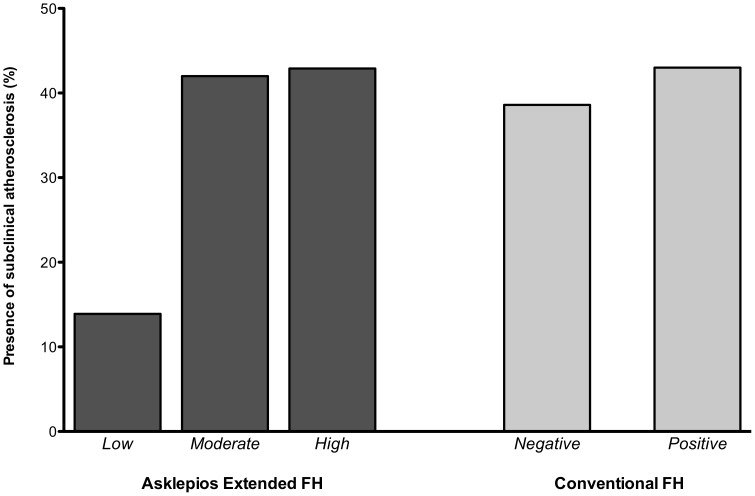
Presence of atherosclerosis according to the proposed new extended family history definition (eFH) and the conventional definition (cFH). Unadjusted data show remarkably less atherosclerosis in the eFH low-risk group (14%) versus the eFH moderate-risk (42%) and eFH high-risk group (43%). There were no large differences observed in prevalence of atherosclerosis when comparing the cFH positive versus negative group (43% versus 39%).

Finally, we assessed the burden of subclinical cardiovascular damage according to both FH definitions. The prevalence of atherosclerosis according to both FH definitions in our study population is presented in Fig. 2.

In age- and sex-adjusted logistic regression analyses, taking the moderate-risk eFH group as the reference category, odds ratios for prevalent atherosclerosis were 0.67 (95% CI 0.51–0.87, p = 0.003) in the low-risk eFH group versus the moderate-risk eFH group. There was no significant increase in prevalent atherosclerosis when comparing eFH high-risk versus moderate-risk categories. For the conventional definition, there was no significant difference on the prevalence of atherosclerosis between cFH positives versus negatives (OR 1.22, 95%CI 0.96–1.54, p = 0.106).

**Figure 3:**
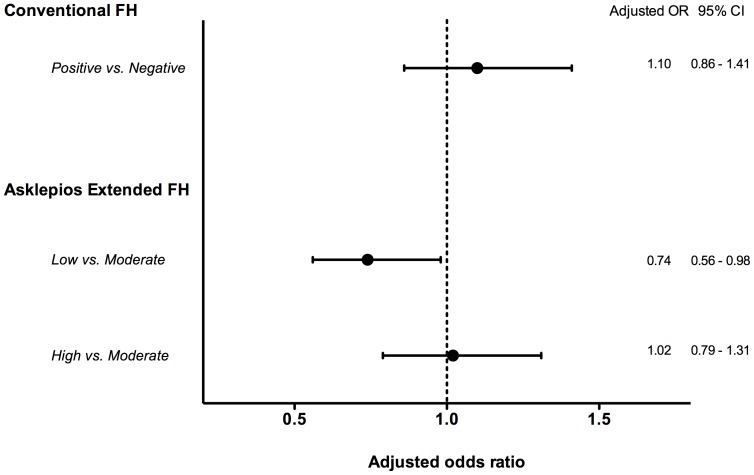
Adjusted odds ratios for presence of subclinical atherosclerosis according to the proposed new extended family history definition (eFH) and the conventional definition (cFH). Odds ratios (95% confidence intervals) for the presence of subclinical atherosclerosis were adjusted for age, sex, total cholesterol, HDL-cholesterol, systolic BP, smoking, DM and BMI. Taking the moderate-risk eFH group as the reference category, odds ratios for prevalent atherosclerosis adjusted for classical risk factors mentioned above are 0.74 (95% CI 0.56–0.98) in the low-risk eFH group versus the moderate-risk eFH group. There was no significant increase in prevalent atherosclerosis when comparing cFH positives versus negatives or eFH high-risk versus moderate-risk categories.

In multivariate adjusted analyses, using classical confounding risk factors (age, sex, total cholesterol, HDL-cholesterol, systolic BP, smoking, BMI and DM), the odds ratios were 0.74 (95% CI 0.56–0.98) in the low-risk eFH group versus the moderate-risk eFH group (Fig. 3). Again, no significant increase in prevalent atherosclerosis was observed when comparing eFH high-risk versus moderate-risk categories or when using the guidelines definition.

We performed sensitivity analyses by 1) analyzing the data for women and men separately, 2) for subjects above and below the age median (45 years) separately, and 3) corrected for educational achievement (as a proxy for social class). We also performed the multivariate analyses by using 1) waist hip ratio and 2) waist circumference instead of BMI as a marker of obesity. The results remained essentially unchanged by these further analyses.

Finally, we tested net reclassification improvement (NRI) and integrated discrimination improvement (IDI) on predicting the presence (or absence) of atherosclerosis by adding the novel eFH definition to a multivariable prediction model including age, sex, systolic blood pressure, total cholesterol, HDL-cholesterol, smoking, BMI and diabetes mellitus in those subjects in the cFH negative ( = eFH low and moderate risk) categories. The continuous NRI was significant (0.217 (95% CI 0.120–0.315), p = 0.00001), the IDI borderline significant (p = 0.068).

## Discussion

In this study, we designed and tested a novel extended family history definition (Asklepios eFH) which: (1) better correlates with metabolic risk factor burden and; (2) independently predicts the presence of subclinical atherosclerosis beyond conventional risk factor burden, unlike cFH assessments. The eFH definition also shows significant improvement in reclassification for the prediction of prevalent atherosclerosis. The new definition differs from the conventional cFH definition in two aspects. First, it includes key additional elements (identified from literature review) so that the new eFH definition also takes into account later occurrence of disease, disease in second-degree relatives (grandparents) and number of affected relatives. Second, the eFH definition divides participants into 3 rather than 2 categories (Fig. 1): a high-risk group, which is almost identical to the guidelines-defined cFH positive group, and a moderate-risk and novel low-risk group, that has a manifestly lower prevalence of subclinical atherosclerosis.

The new Asklepios eFH definition is superior in detecting adverse CVD risk profiles in the general population. It exposes a significantly greater differential in risk profile than cFH. The eFH definition (but not the cFH definition) was additionally associated with unfavourable glycemic and lipid profiles, more components of the metabolic syndrome and more atherosclerosis. Furthermore, an important novel finding is that most relations with eFH were not graded but showed clear informational breakpoints with the eFH low-risk group being particularly interesting. Much of the additional information extracted by the novel eFH definition can be attributed to the presence of this newly defined low-risk category. The cFH positive group, the eFH high-risk and large eFH moderate-risk groups have quite similar risk factor profiles and preclinical atherosclerotic burdens. Separating out the low-risk category in many cases abolished a large part of the step-up in adverse risk profiles found in the cFH positive group, suggesting that the differences between the guidelines positive and negative groups are (in part) due to the admixture of the eFH low-risk group to the latter (i.e. some differences are due to a significantly better risk profile in the eFH low-risk group).

Most importantly, the new eFH definition (but not the conventional definition) was able to identify presence of atherosclerosis beyond conventional risk factor burden, indicating that FH conveys additional information, not completely characterized by simply measuring a risk factor profile.

A few studies already showed the value of extending FH beyond a simple yes/no question about presence of disease in a first-degree relative [10], [12], [14]. Scheuner et al. investigated various binary definitions of FH and found significant associations between a personal history of CHD and an additional FH that goes beyond having first-degree relatives with early-onset CHD [12]. In line with their work we took into account key additional elements in our new eFH definition, and we elaborated on their dichotomous definitions by defining a three-tier definition encompassing both the classic high-risk group, as well as a novel and highly interesting low-risk group.

That presence of a positive FH is associated with an adverse risk profile is largely in agreement with published reports [30], [31]. This familial aggregation of cardiovascular risk factors reflects the genetic and environmental influence on the causal pathways of familial CVD. In line with published data we found an association with fibrinogen (a thrombotic risk factor), but not with hs-CRP, for which literature is inconsistent [32], [33], [34], [35], [36].

Regarding the presence of atherosclerosis (a prognostically well-validated non-invasive surrogate endpoint to assess cardiovascular risk), the eFH low-risk group had significantly less atherosclerosis compared to the eFH moderate- and high-risk groups. Although other studies already demonstrated significantly more atherosclerosis in patients with a FH of CVD [37], [38]. Our study shows, for the first time, that this is in large part due to significantly less atherosclerosis in a “low-risk” group that can be readily identified by eFH (but not by cFH), separating it from an intermediate group (which would conventionally be classified as having a negative FH). The eFH moderate risk group demonstrates almost identical prevalence of atherosclerosis as the cFH positive group (corresponding to the eFH high-risk group). This large eFH moderate-risk group with a substantial atherosclerotic burden would be overlooked when using the conventional FH definition.

### Clinical Relevance

Although FH is an important risk factor for CVD and plays an important role in medical practice, it is underused in CVD prevention efforts [8], [39]. Recent data from a randomized controlled trial looking at the added value and feasibility of systematically collecting FH, found that it increases the proportion of persons identified as having high cardiovascular risk for further targeted prevention (many risk factors are amenable to intervention, by lifestyle or pharmacologically) [40], [41]. FH also received increased visibility as a risk qualifier in the new European guidelines on CVD prevention, where a positive cFH is considered to increase the 10-year risk of a fatal CV by 1.7-fold in women and by 2.0-fold in men [42]. Conversely, it is suggested that 10-year CVD risk (SCORE) may be lower in those with a FH of longevity. Whilst knowledge of FH may not affect clinical decision making in those at very high or very low predicted risk, it may aid in discriminating risk among the very large group of subjects at intermediate levels of predicted risk [43]. Since low-risk populations for CVD are gaining interest, our eFH low-risk group with a more favourable risk profile and significantly less atherosclerosis, could be an interesting phenotype for further study [44]. Yeboah et al. recently studied novel risk markers, comprising (premature and non-premature) FH of CHD in a first-degree relative, for improvement in cardiovascular risk assessment in intermediate-risk individuals. The authors found that FH was an independent predictor of CHD/CVD in intermediate-risk individuals. Furthermore, besides coronary calcium score, FH performed the best for CHD risk reclassification (NRI = 0.160). Interestingly, most of the correct reclassification was based on subjects reclassified into a lower risk category [45].

Intuitively, it seems likely that shared lifestyle risk factors (smoking, diet, physical inactivity) represent (non-genetic) pathways through which FH influences risk of CVD. The literature is inconsistent [36], [46]; we found no clear associations between FH and lifestyle parameters. It is possible that increased perception of familial risk does not automatically lead to changed behaviour; some people may even adopt a fatalistic outlook and make no efforts at all to decrease their risk [47].

### Study Strengths and Limitations

The major limitation of the present study is the absence of outcome data. We used a surrogate measure that is well validated as a prognostic marker: presence of atherosclerosis. Furthermore, it remains to be tested if our findings can be extrapolated to the much younger (where relatives might not have aged sufficiently to have suffered CV events) or older populations. One of the major problems of defining and studying FH is that it is a “moving target”. A 52-year old man today that is eFH low-risk could end-up tomorrow in the eFH high-risk category after his brother had a myocardial infarction. Reassuringly, sensitivity analyses in our cohort looking at the older and younger subjects (with correspondingly older/younger family members) showed similar results. A major strength is the population-based nature of the study, combining a well-balanced, representative sample with stringent methodology, and a broad and detailed array of carefully assessed cardiovascular intermediate phenotypes.

Self-reported FH of CVD was not validated through medical records, which is another potential limitation. Many, though not all, previous studies showed that questionnaires considering FH of CVD can be considered as accurate and people can correctly report their FH for CVD [15], [48], [49], [50], [51]. Moreover, our assessment of FH through self-report is similar to general practice, thus the present findings can be generalized to the usual clinical setting. We used a categorical definition rather than a (theoretically attractive) continuous FH score, where entry is restricted to more informative (i.e. larger) families and a single affected family member is ruled out [50]. Considering the characteristics of our study population families (average European family size), we followed the recommendations from Silberberg et al., who previously recommended that the use of categorical definitions are more likely to be adequate in smaller families and few affected relatives [52]. Finally, 13.6% of subjects could not be correctly classified because of insufficiently accurate knowledge of their FH.

### Conclusion

In this study, we designed and tested a novel extended family history definition (Asklepios eFH) which: (1) better correlates with metabolic risk factor burden and (2) independently predicts the presence of subclinical atherosclerosis beyond conventional risk factor burden, unlike cFH assessments and (3) shows significant improvement in reclassification for the prediction of prevalent atherosclerosis. Adding information on non-first degree relatives, late occurrence of disease and number of affected relatives to the FH construct improves the discrimination for cardiovascular risk factors and atherosclerotic burden in order to better target individuals for CVD prevention efforts. The new eFH definition separates the cFH negative group into two categories: a large eFH moderate-risk group, and a smaller eFH low-risk group. The latter is a particularly interesting phenotype for further study, having a more favourable risk profile and significantly less atherosclerosis (odds ratio 0.74). There remain clear opportunities to refine and increase the performance and informational content of this readily available, simple, inexpensive tool.

## Supporting Information

Questionnaire S1 **The Asklepios Family History (FH) Questionnaire.** The Asklepios FH Questionnaire was created specifically for this study. It provides data on the occurrence of cardiovascular disease (CVD) in 4 generations of the respondent’s genetic family (parents, grandparents, siblings and offspring). For all family members, respondents provided the year of birth and the year and cause of death. The questionnaire further queries for the occurrence of fatal and nonfatal CVD events: myocardial infarction, coronary revascularisation, peripheral vascular intervention of inguinal or lower limb arteries, stroke, carotid revascularisation or sudden cardiac death.
(PDF)

## References

[1] Andresdottir MB, Sigurdsson G, Sigvaldason H, Gudnason V (2002) Fifteen percent of myocardial infarctions and coronary revascularizations explained by family history unrelated to conventional risk factors. The Reykjavik Cohort Study. Eur Heart J 23: 1655–1663. 12398822 10.1053/euhj.2002.3235

[2] Barrett-Connor E, Khaw K (1984) Family history of heart attack as an independent predictor of death due to cardiovascular disease. Circulation 69: 1065–1069. 6713610 10.1161/01.cir.69.6.1065

[3] Friedlander Y, Arbogast P, Schwartz SM, Marcovina SM, Austin MA, et al (2001) Family history as a risk factor for early onset myocardial infarction in young women. Atherosclerosis 156: 201–207. 11369015 10.1016/s0021-9150(00)00635-3

[4] Heller RF, Kelson MC (1983) Family history in "low risk" men with coronary heart disease. J Epidemiol Community Health 37: 29–31. 6875441 10.1136/jech.37.1.29 PMC1052251

[5] Wilson BJ, Qureshi N, Santaguida P, Little J, Carroll JC, et al (2009) Systematic review: family history in risk assessment for common diseases. Ann Intern Med 151: 878–885. 19884616 10.7326/0003-4819-151-12-200912150-00177

[6] Frezzo TM, Rubinstein WS, Dunham D, Ormond KE (2003) The genetic family history as a risk assessment tool in internal medicine. Genet Med 5: 84–91. 12644777 10.1097/01.GIM.0000055197.23822.5E

[7] Assimes TL (2011) Family history of heart disease: the re-emergence of a traditional risk factor. J Am Coll Cardiol 57: 628–629. 21272755 10.1016/j.jacc.2010.09.036

[8] Banerjee A (2012) A review of family history of cardiovascular disease: risk factor and research tool. Int J Clin Pract 66: 536–543. 22607505 10.1111/j.1742-1241.2012.02908.x

[9] Berg AO, Baird MA, Botkin JR, Driscoll DA, Fishman PA, et al (2009) National Institutes of Health State-of-the-Science Conference Statement: Family History and Improving Health. Ann Intern Med 151: 872–877. 19884615 10.7326/0003-4819-151-12-200912150-00165

[10] Silberberg JS, Wlodarczyk J, Fryer J, Robertson R, Hensley MJ (1998) Risk associated with various definitions of family history of coronary heart disease. The Newcastle Family History Study II. Am J Epidemiol 147: 1133–1139. 9645791 10.1093/oxfordjournals.aje.a009411

[11] Graham I, Atar D, Borch-Johnsen K, Boysen G, Burell G, et al (2007) European guidelines on cardiovascular disease prevention in clinical practice: full text. Fourth Joint Task Force of the European Society of Cardiology and other societies on cardiovascular disease prevention in clinical practice (constituted by representatives of nine societies and by invited experts). Eur J Cardiovasc Prev Rehabil 14 Suppl 2S1–113. 10.1097/01.hjr.0000277983.23934.c9 17726407

[12] Scheuner MT, Whitworth WC, McGruder H, Yoon PW, Khoury MJ (2006) Expanding the definition of a positive family history for early-onset coronary heart disease. Genet Med 8: 491–501. 16912580 10.1097/01.gim.0000232582.91028.03

[13] Sesso HD, Lee IM, Gaziano JM, Rexrode KM, Glynn RJ, et al (2001) Maternal and paternal history of myocardial infarction and risk of cardiovascular disease in men and women. Circulation 104: 393–398. 11468199 10.1161/hc2901.093115

[14] Scheuner MT, Setodji CM, Pankow JS, Blumenthal RS, Keeler E (2008) Relation of familial patterns of coronary heart disease, stroke, and diabetes to subclinical atherosclerosis: the multi-ethnic study of atherosclerosis. Genet Med 10: 879–887. 19092440 10.1097/GIM.0b013e31818e639b PMC2684807

[15] Murabito JM, Pencina MJ, Nam BH, D'Agostino RB Sr, Wang TJ, et al (2005) Sibling cardiovascular disease as a risk factor for cardiovascular disease in middle-aged adults. JAMA 294: 3117–3123. 16380592 10.1001/jama.294.24.3117

[16] Nasir K, Michos ED, Rumberger JA, Braunstein JB, Post WS, et al (2004) Coronary artery calcification and family history of premature coronary heart disease: sibling history is more strongly associated than parental history. Circulation 110: 2150–2156. 15466626 10.1161/01.CIR.0000144464.11080.14

[17] Kinra S, Davey Smith G, Okasha M, McCarron P, McEwen J (2003) Is maternal transmission of coronary heart disease risk stronger than paternal transmission? Heart 89: 834–838. 12860850 10.1136/heart.89.8.834 PMC1767769

[18] Rietzschel ER, De Buyzere ML, Bekaert S, Segers P, De Bacquer D, et al (2007) Rationale, design, methods and baseline characteristics of the Asklepios Study. Eur J Cardiovasc Prev Rehabil 14: 179–191. 17446795 10.1097/HJR.0b013e328012c380

[19] Grundy SM, Cleeman JI, Daniels SR, Donato KA, Eckel RH, et al (2005) Diagnosis and management of the metabolic syndrome: an American Heart Association/National Heart, Lung, and Blood Institute Scientific Statement. Circulation 112: 2735–2752. 16157765 10.1161/CIRCULATIONAHA.105.169404

[20] Wong LS, Huzen J, de Boer RA, van Gilst WH, van Veldhuisen DJ, et al (2011) Telomere length of circulating leukocyte subpopulations and buccal cells in patients with ischemic heart failure and their offspring. PloS one 6: e23118. 21876736 10.1371/journal.pone.0023118 PMC3158078

[21] Langlois MR, Rietzschel ER, De Buyzere ML, De Bacquer D, Bekaert S, et al (2008) Femoral plaques confound the association of circulating oxidized low-density lipoprotein with carotid atherosclerosis in a general population aged 35 to 55 years: the Asklepios Study. Arterioscler Thromb Vasc Biol 28: 1563–1568. 18511698 10.1161/ATVBAHA.108.167346

[22] Rietzschel ER, Langlois M, De Buyzere ML, Segers P, De Bacquer D, et al (2008) Oxidized low-density lipoprotein cholesterol is associated with decreases in cardiac function independent of vascular alterations. Hypertension 52: 535–541. 18663154 10.1161/HYPERTENSIONAHA.108.114439

[23] Touboul PJ, Hennerici MG, Meairs S, Adams H, Amarenco P, et al (2007) Mannheim carotid intima-media thickness consensus (2004–2006). An update on behalf of the Advisory Board of the 3rd and 4th Watching the Risk Symposium, 13th and 15th European Stroke Conferences, Mannheim, Germany, 2004, and Brussels, Belgium, 2006. CerebrovascDis 23: 75–80. 10.1159/000097034 17108679

[24] Vermeersch S, Rietzschel E, De Buyzere M, Van Bortel L, D'asseler Y, et al (2007) Validation of a new automated IMT measurement algorithm. J Hum Hypertens 21: 976–978. 17568751 10.1038/sj.jhh.1002251

[25] De Meyer T, Van daele CM, De Buyzere ML, Denil SL, De Bacquer D, et al (2012) No shorter telomeres in subjects with a family history of cardiovascular disease in the Asklepios study. Arterioscler Thromb Vasc Biol 32: 3076–3081. 23087363 10.1161/ATVBAHA.112.300341

[26] Scheuner MT, Wang SJ, Raffel LJ, Larabell SK, Rotter JI (1997) Family history: a comprehensive genetic risk assessment method for the chronic conditions of adulthood. Am J Med Genet 71: 315–324. 9268102 10.1002/(sici)1096-8628(19970822)71:3<315::aid-ajmg12>3.0.co;2-n

[27] Kundu S, Aulchenko YS, van Duijn CM, Janssens AC (2011) PredictABEL: an R package for the assessment of risk prediction models. Eur J Epidemiol 26: 261–264. 21431839 10.1007/s10654-011-9567-4 PMC3088798

[28] Pencina MJ, D'Agostino RB Sr, D'Agostino RB Jr, Vasan RS (2008) Evaluating the added predictive ability of a new marker: from area under the ROC curve to reclassification and beyond. Stat Med 27: 157–172; discussion 207–212. 10.1002/sim.2929 17569110

[29] Pencina MJ, D'Agostino RB Sr, Steyerberg EW (2011) Extensions of net reclassification improvement calculations to measure usefulness of new biomarkers. Stat Med 30: 11–21. 21204120 10.1002/sim.4085 PMC3341973

[30] Scheuner MT, Whitworth WC, McGruder H, Yoon PW, Khoury MJ (2006) Familial risk assessment for early-onset coronary heart disease. Genet Med 8: 525–531. 16912584 10.1097/01.gim.0000232480.00293.00

[31] Chow CK, Islam S, Bautista L, Rumboldt Z, Yusufali A, et al (2011) Parental history and myocardial infarction risk across the world: the INTERHEART Study. J Am Coll Cardiol 57: 619–627. 21272754 10.1016/j.jacc.2010.07.054

[32] Mills JD, Mansfield MW, Grant PJ (2002) Tissue plasminogen activator, fibrin D-dimer, and insulin resistance in the relatives of patients with premature coronary artery disease. Arterioscler Thromb Vasc Biol 22: 704–709. 11950714 10.1161/hq0402.105902

[33] Pankow JS, Folsom AR, Province MA, Rao DC, Eckfeldt J, et al (1997) Family history of coronary heart disease and hemostatic variables in middle-aged adults. Atherosclerosis Risk in Communities Investigators and Family Heart Study Research Group. Thromb Haemost 77: 87–93. 9031455

[34] Waters D (2010) Risk Factors for Cardiovascular Disease. In: MH Crawford JD, WJ Paulus, editors. Cardiology. 3 ed. Philadelphia: Mosby Elsevier.

[35] Ford ES, Giles WH, Mokdad AH (2005) Family history of diabetes or cardiovascular disease and C-reactive protein concentration: findings from the National Health and Nutrition Examination Survey, 1999–2000. Am J Prev Med 29: 57–62. 16389127 10.1016/j.amepre.2005.07.018

[36] Hamer M, Chida Y, Stamatakis E (2009) The role of conventional and novel mechanisms in explaining increased risk of cardiovascular events in offspring with positive parental history. J Hypertens 27: 1966–1971. 19587606 10.1097/HJH.0b013e32832f0d6f

[37] Gaeta G, De Michele M, Cuomo S, Guarini P, Foglia MC, et al (2000) Arterial abnormalities in the offspring of patients with premature myocardial infarction. N Engl J Med 343: 840–846. 10995863 10.1056/NEJM200009213431203

[38] de Giorgis T, Giannini C, Scarinci A, D'Adamo E, Agostinelli S, et al (2009) Family history of premature cardiovascular disease as a sole and independent risk factor for increased carotid intima-media thickness. J Hypertens 27: 822–828. 19516181 10.1097/HJH.0b013e328325d81b

[39] Perk J, De Backer G, Gohlke H, Graham I, Reiner Z, et al (2012) European Guidelines on cardiovascular disease prevention in clinical practice (version 2012): The Fifth Joint Task Force of the European Society of Cardiology and Other Societies on Cardiovascular Disease Prevention in Clinical Practice (constituted by representatives of nine societies and by invited experts) * Developed with the special contribution of the European Association for Cardiovascular Prevention & Rehabilitation (EACPR). Eur Heart J 33: 1635–701. 22555213 10.1093/eurheartj/ehs092

[40] Qureshi N, Armstrong S, Dhiman P, Saukko P, Middlemass J, et al (2012) Effect of adding systematic family history enquiry to cardiovascular disease risk assessment in primary care: a matched-pair, cluster randomized trial. Ann Intern Med 156: 253–262. 22351711 10.7326/0003-4819-156-4-201202210-00002

[41] Berg AO (2012) Family history gets a boost. Ann Intern Med 156: 315–316. 22351717 10.7326/0003-4819-156-4-201202210-00012

[42] Viera AJ, Garrett JM (2005) Understanding interobserver agreement: the kappa statistic. Fam Med 37: 360–363. 15883903

[43] Lloyd-Jones DM, Nam BH, D'Agostino RB Sr, Levy D, Murabito JM, et al (2004) Parental cardiovascular disease as a risk factor for cardiovascular disease in middle-aged adults: a prospective study of parents and offspring. JAMA 291: 2204–2211. 15138242 10.1001/jama.291.18.2204

[44] Gabioud A, Waeber G, Vollenweider P, Marques-Vidal P (2012) Who is at low risk for cardiovascular disease? An assessment of different definitions. Int J Cardiol. In press. 10.1016/j.ijcard.2012.07.004 22882961

[45] Yeboah J, McClelland RL, Polonsky TS, Burke GL, Sibley CT, et al (2012) Comparison of novel risk markers for improvement in cardiovascular risk assessment in intermediate-risk individuals. JAMA 308: 788–795. 22910756 10.1001/jama.2012.9624 PMC4141475

[46] Elis A, Pereg D, Tirosh A, Shochat T, Tekes-Manova D, et al (2008) Family history of cardiovascular disease does not predict risk-reducing behavior. Eur J Cardiovasc Prev Rehabil 15: 325–328. 18525388 10.1097/HJR.0b013e3282f50ed8

[47] Kip KE, McCreath HE, Roseman JM, Hulley SB, Schreiner PJ (2002) Absence of risk factor change in young adults after family heart attack or stroke: the CARDIA Study. Am J Prev Med 22: 258–266. 11988382 10.1016/s0749-3797(02)00416-6

[48] Bensen JT, Liese AD, Rushing JT, Province M, Folsom AR, et al (1999) Accuracy of proband reported family history: the NHLBI Family Heart Study (FHS). Genet Epidemiol 17: 141–150. 10414557 10.1002/(SICI)1098-2272(1999)17:2<141::AID-GEPI4>3.0.CO;2-Q

[49] Silberberg JS, Wlodarczyk J, Fryer J, Ray CD, Hensley MJ (1998) Correction for biases in a population-based study of family history and coronary heart disease. The Newcastle Family History Study I. Am J Epidemiol 147: 1123–1132. 9645790 10.1093/oxfordjournals.aje.a009410

[50] Williams RR, Hunt SC, Heiss G, Province MA, Bensen JT, et al (2001) Usefulness of cardiovascular family history data for population-based preventive medicine and medical research (the Health Family Tree Study and the NHLBI Family Heart Study). Am J Cardiol 87: 129–135. 11152826 10.1016/s0002-9149(00)01303-5

[51] De Backer G, Hulstaert F, De Munck K, Rosseneu M, Van Parijs L, et al (1986) Serum lipids and apoproteins in students whose parents suffered prematurely from a myocardial infarction. Am Heart J 112: 478–484. 3092607 10.1016/0002-8703(86)90510-7

[52] Silberberg J, Fryer J, Wlodarczyk J, Robertson R, Dear K (1999) Comparison of family history measures used to identify high risk of coronary heart disease. Genet Epidemiol 16: 344–355. 10207716 10.1002/(SICI)1098-2272(1999)16:4<344::AID-GEPI2>3.0.CO;2-Q

