# Supplementary material for: Addition of a Novel, Protective Family History Category Allows Better Profiling of Cardiovascular Risk and Atherosclerotic Burden in the General Population. The Asklepios Study
Source: PLoS One. 2013 May 2;8(5):e63185. doi: 10.1371/journal.pone.0063185 (PMC3642069; doi:10.1371/journal.pone.0063185)
Supplement: Questionnaire S1 — The Asklepios Family History (FH) Questionnaire. The Asklepios FH Questionnaire was created specifically for this study. It provides data on the occurrence of cardiovascular disease (CVD) in 4 generations of the respondent’s genetic family (parents, grandparents, siblings and offspring). For all family members, respondents provided the year of birth and the year and cause of death. The questionnaire further queries for the occurrence of fatal and nonfatal CVD events: myocardial infarction, coronary revascularisation, peripheral vascular intervention of inguinal or lower limb arteries, stroke, carotid revascularisation or sudden cardiac death. (PDF) [file pone.0063185.s001.pdf]

**YOUR FATHER** *(UNIQUE IDENTIFIER)*

E1. Date of birth (year) [ ]

E2. Is this person still alive?
- ☐ yes, he lives in [ ]
- ☐ no, he died in (year) [ ]

E3. Cause of death (if applicable)?

[ ]

E4. Did he ever experience one of the following? (multiple items possible)

| | which year? | | cause of death? |
|---|---|---|---|
| ☐ acute myocardial infarction or heart attack | [ ] | [ ] | ☐ yes ☐ no |
| ☐ coronary revascularisation (PCI, CABG) | [ ] | [ ] | ☐ yes ☐ no |
| ☐ peripheral artery revascularisation (femoral or lower limb) | [ ] | [ ] | ☐ yes ☐ no |
| ☐ stroke / cerebrovascular accident | [ ] | [ ] | ☐ yes ☐ no |
| ☐ carotid revascularisation | [ ] | [ ] | ☐ yes ☐ no |
| ☐ sudden death | [ ] | [ ] | |
| ☐ none of the above | | | |
| ☐ I don't know | | | |

E5. Did he ever had one of the following risk factors? (multiple items possible)

- ☐ hypertension
- ☐ hypercholesterolemia
- ☐ diabetes mellitus
- ☐ obesity
- ☐ smoking (more than one year)
- ☐ none of the above
- ☐ I don't know
